# Supplementary material for: Emergency Craniotomy and Burr-Hole Trephination in a Low-Resource Setting: Capacity Building at a Regional Hospital in Cambodia
Source: Int J Environ Res Public Health. 2022 May 26;19(11):6471. doi: 10.3390/ijerph19116471 (PMC9179964; doi:10.3390/ijerph19116471)
Supplement: Supplementary file 1 [file ijerph-19-06471-s001.zip › ijerph-1734342-supplementary.pdf]

Supplementary Table S1. Baseline characteristics of the study population grouped by preoperative GCS score.

Quantitative variables	All (n = 235)			Group 1: Preoperative GCS ≥ 7 (n = 198)			Group 2: Preoperative GCS < 7 (n = 37)			p-Value
	N (Missing)	Mean (SD)	Median (Min–Max)	N (Missing)	Mean (SD)	Median (Min–Max)	N (Missing)	Mean (SD)	Median (Min–Max)	
**Age**	235 (0)	34.9 (17.3)	28 (12-84)	198 (0)	35.2 (17.5)	29 (12-84)	37 (0)	33.5 (16.1)	26 (15-69)	0.647 [a]
**Time from injury to admission (hours)**	234 (1)	83 (213.5)	24 (1-2160)	197 (1)	90.8 (227.4)	24 (1-2160)	37 (0)	41.4 (107)	24 (2-576)	0.553 [a]
**Time from admission to surgery (hours)**	235 (0)	21.4 (36.4)	8 (1-336)	198 (0)	22.4 (38.9)	9 (1-336)	37 (0)	16.1 (17.9)	7 (3-72)	0.499 [a]
**ISS score**	235 (0)	23.2 (3.6)	25 (16-25)	198 (0)	23.1 (3.7)	25 (16-25)	37 (0)	24 (2.8)	25 (16-25)	0.144 [a]
**Admission GCS**	235 (0)	9.9 (2.8)	10 (3-15)	198 (0)	10.8 (2.2)	11 (7-15)	37 (0)	5.5 (1.1)	6 (3-8)	< 0.001 [a]
**Preoperative GCS**	235 (0)	9.8 (2.8)	10 (3-15)	198 (0)	10.7 (2.2)	11 (7-15)	37 (0)	5.3 (0.8)	5 (3-6)	< 0.001 [a]

Categorical variables	Category	All (n = 235)		Group 1: Preoperative GCS ≥ 7 (n = 198)		Group 2: Preoperative GCS < 7 (n = 37)		p-Value
		Count	Percentage	Count	Percentage	Count	Percentage	
**Age**	< 20y/ 20-34y/ 35-49y/ 50-64y/ ≥ 65y	41/105/29/40/20	17.4/44.7/12.3/17/8.5	36/86/24/34/18	18/43/12/17/9	5/19/5/6/2	14/51/14/16/5	0.889 [c]
**Head injury severity**	Severe/Moderate/Mild	83/101/51	35.3/43/21.7	46/101/51	23.3/51/25.8	37/0/0	100/0/0	< 0.001 [b]
**Gender**	Female/Male	29/206	12.3/87.7	24/174	12.1/87.9	5/32	13.5/86.5	0.788 [c]
**Type of fracture**	Close fracture/Open fracture/Without fracture	130/8/97	55.3/3.4/41.3	108/8/82	54.5/4.0/41.4	22/0/15	59.5/0.0/40.5	0.441 [b]
**Trauma diagnosis**	Epidural hematoma/Subdural hematoma/Intracerebral hematoma/Chronic	100/74/35/26	42.6/31.5/14.9/11.1	87/62/26/23	43.9/31.3/13.1/11.6	13/12/9/3	35.1/32.4/24.3/8.1	0.314 [b]
**Referral admission**	No/Yes	88/147	37.4/62.6	72/126	36.4/63.6	16/21	43.2/56.8	0.427 [b]
**Polytrauma**	No other injuries/Other moderate injuries with no need for surgery/Other injuries with the need for surgery	68/162/5	28.9/68.9/2.1	61/132/5	30.8/66.7/2.5	7/30/0	18.9/81.1/0.0	0.238 [a]
**Surgery type**	Burr-hole trephination /Craniotomy	28/207	11.9/88.1	25/173	12.6/87.4	3/34	8.1/91.9	0.585 [c]

Abbreviations: GCS, Glasgow Coma Scale; ISS, Injury Severity Score.

[a] Mann–Whitney U test.

[b] Chi-Square test.

[c] Fisher’s Exact test if more than 20% cells have expected count less than 5 in Chi-Square test.
